# Design of robust cubature fission particle filter algorithm in multi-source cooperative navigation

**DOI:** 10.1038/s41598-022-08189-x

**Published:** 2022-03-10

**Authors:** Wei Sun, Jingzhou Liu

**Affiliations:** School of Geomatics, Liaoning Technical University, Fuxin, 123000 Liaoning China

**Keywords:** Aerospace engineering, Electrical and electronic engineering

## Abstract

As a part of the multi-source cooperative navigation scheme, data fusion has a significant impact on the quality of state estimation. Particle filtering has gradually become the focus of many fusion methods due to its unique theoretical advantages in nonlinear non-Gaussian systems. However, the particle degradation and the resulting sample impoverishment restrict its application in complex engineering scenarios. In this paper, a robust cubature fission particle filter (RCFPF) is proposed to deal with these problems. First, in the framework of cubature rule, Huber function is used to combine the L2 norm and L1 norm to improve the importance density function(IDF), suppress the observation noise. Meanwhile, the proposed distribution(PD) is further optimized by combining the Gaussian distribution with Laplace distribution to alleviate particle degradation. Second, the particle swarm is fissioned before resampling, and the particle weight is reconstructed by fission of high weight particles and covering low weight particles to inhibit sample impoverishment. The vehicle experiments of multi-source cooperative navigation show that the proposed algorithm achieves better test results in accuracy and robustness than extended Kalman filter (EKF), strong tracking particle filter (STPF), and cubature particle filter (CPF).

## Introduction

The relative position provided by cooperative navigation is the basis of vehicle intelligent transportation and location-based service^1,2^, which can meet a variety of road traffic requirements, including smart transportation, collision avoidance, and congestion reduction. Therefore, it has attracted extensive attention in recent years. In order to improve the efficiency of position perception and obtain higher precision cooperative navigation solutions, scholars have made a lot of research work and gradually improve the quality of navigation parameters^3–7^. However, most of these studies integrate new navigation modes from the perspective of measurement and update the cooperative navigation system to obtain better solutions. There are few improvements on the data fusion methods that play an important role in the solution. The improvement of relevant literature remains within the framework of Kalman filter (KF) requiring linear Gaussian system^8^. Because of the inherent theoretical deficiency, its accuracy in dealing with multi-source complex systems is always limited to some extent. Unlike Kalman filter, particle filter (PF) has almost no requirements for the system and noise characteristics^9^, so it becomes a possibility of multi-source cooperative navigation data fusion.

As one of the realization methods of Markov-Bayesian recursion, PF uses a set of weighted particles to iteratively update to approximate a posteriori probability density distribution of the system model. In theory, it can carry out probability reasoning and recursive estimation for any system^10^. Gordon et al. performed resampling after Monte Carlo importance sampling, so that PF began to have a basis for practical applications^11^. Since then, PF has gradually become a focus in the field of data fusion and improved with various theories^12–18^. Although particle filtering has made great progress in recent years, particle degradation and the resulting sample impoverishment problem have always restricted the further development and application of the above-mentioned various improved PFs^12–18^. Particle degradation means that after multiple iterations, only a small number of particles in the particle swarm have large weights, while most of the particles have negligible weights, so that a lot of calculation time and resources are wasted on particles with small weights, which affects the performance of the algorithm^19–21^. Kong has proved that particle degradation is an inherent problem of PF^22^. Doucet and Wang further pointed out that the variance of particle weight increases with time^23,24^. However, the proposed distribution (PD) generated by the importance density function (IDF) in PF is the direct approximation of the particle to the posterior probability distribution, and its simulation accuracy has an important impact on the estimation performance. Selecting an appropriate IDF can suppress the weight variance and alleviate the degradation problem^25^. Therefore, researchers have carried out a lot of researches around IDF.

The state equation is the earliest and most extensive choice of IDF because of its relative simplicity and easy accessibility^26^. This choice is easy to implement and will not increase additional computational load. However, using the state transition probability density represented by the state equation as the PD does not consider the current measurement information. Since the particle weight is proportional to the likelihood function, choosing the equation of state as the IDF will make the variance of the particle weight larger, especially when the likelihood function is relatively "steep" or located at the tail of the state transition probability distribution, it will accelerate the degradation and even cause the filter divergence^27^. To solve this problem, related scholars introduced the annealing algorithm to optimize the probability density^28,29^. Although the number of particles in the likelihood region is increased, the loop process introduced by the annealing optimization also greatly increases the computational load and limits the practical application of PF. Another idea of IDF selection is to introduce measurement, i.e., using existing filtering methods to make the a priori distribution and the likelihood function have a larger overlap area through the measurement so as to better match the posterior distribution. According to different Bayesian integral approximation strategies, commonly used improved PF includes extended particle filter (EPF)^30^ based on EKF, unscented particle filter (UPF) based on Unscented Kalman filter (UKF)^31^, and cubature particle filter (CPF) based on cubature Kalman filter (CKF)^32^. Since the use of existing filtering as IDF can greatly improve the calculation performance of PF under the condition of appropriately increasing the amount of calculation, this method is more and more favored by researchers and has achieved considerable development. Liu and Hu have obtained a strong tracking particle filter (STPF) based on the improved EKF, which was used to compensate the model error and apply it to fault detection^33,34^. Zhang uses the truncated adaptive theory to generate the proposed distribution under the CPF framework to optimize the accuracy of the algorithm in strongly nonlinear systems^35^. Havangi uses a particle swarm optimization algorithm to improve UKF, adjust the position and velocity of the particle swarm to improve the accuracy of the posterior probability of particle simulation^36^.

However, the various studies mentioned above all use different methods to improve the KF to produce PD to approximate the posterior probability density within the PF framework. Although its accuracy is continuously improved through the combination of various theories, the theoretical basis of these methods are based on the central limit theorem and limited to the Gaussian distribution of L2 norm estimation. The fusion of the L1 norm only revolves around its sparsity and fails to make full use of other properties of the L1 norm^16–18^. In fact, for more complex multi-source systems, the posterior distribution is often affected by multiple factors, which is difficult to approximate it with a single distribution as "irregular". The definition of PD in Gaussian distribution limits the superiority of PF in dealing with general nonlinear non-Gaussian systems. At the same time, various studies focus on the approximation of the real model of the system and few literatures pay a attention to suppressing the interference of various noises of the sensor itself to the PD.

In this paper, for the above-mentioned deficiencies and limitations the Huber M estimation based on the third-order cubature law is applied to generate PD in PF. After the cubature rule is applied to transfer the system states, the algorithm combines the L2 norm and L1 norm using the Huber function for a better approximation of the posterior probability distribution. The posterior probability distribution of the system is redefined through their corresponding Gaussian distribution and Laplace distribution. The proposed idea also can make full use of the robustness of the L1 norm to suppress the interference of observation noise to PD. In order to further deal with the sample impoverishment problem, the algorithm uses the fission method to derive the high weight particles before resampling, and uses the offspring particles to cover the low weight particles in the parent to reconstruct the particle swarm weight. Finally, the robust cubature fission particle filter is formed. The vehicle experiments of multi-source cooperative navigation show that RCFPF performs better in accuracy and robustness than EKF, STPF, and CPF. It provides a new idea for optimizing PD, alleviating particle degradation and sample impoverishment, and also provides a new algorithm for data fusion of multi-source cooperative navigation.

The rest of the paper is organized as follows: the second part introduces the cooperative navigation system, mainly the measurement of each subsystem that needs data fusion. The third part introduces the specific methods of the algorithm, including the process of classical PF, the construction of IDF and the specific process of particle swarm weight reconstruction. In the fourth part, the vehicle experiment of cooperative navigation is carried out, then the results comparison and analysis are given. The fifth part summarizes the paper and gives the conclusion.

## Cooperative navigation

Vehicle cooperative navigation is generally realized on the platform of vehicle ad hoc networks (VANET). In order to simplify the system and facilitate interpretation, this paper takes the working logic between vehicles *a* and *b* (receiver *a* and receiver *b*) in VANET as an example, and its algorithm and conclusion can be extended to ad hoc networks with more vehicles^7^. In this article, the cooperative positioning system uses the pseudo range and Doppler frequency shift acquired by the receiver, the relative distance measured by Ultra Wide Band(UWB) to realize it. The workflow of relative position sensing of cooperative navigation is shown as follows: vehicle *a* obtains additional information through UWB communication, i.e., the pseudorange and Doppler frequency shift of vehicle *b*, makes double difference in combination with local pseudorange and Doppler frequency shift, measures the distance between two vehicles with UWB, and performs data fusion of the navigation system with the help of RCFPF to obtain the final cooperative navigation positioning solution. In order to fully demonstrate the algorithm process, we first introduces the fusion measurements required by each navigation system. Second, the system equations of cooperative navigation are given, and the data fusion is carried out in its framework.

### GPS observation

As one of the most important observations of the receiver, the pseudorange can be used to determine position information through single-point positioning, i.e., three-dimensional coordinates and time can be calculated through the pseudorange of at least four satellites. In tightly integrated cooperative navigation, the system only obtains pseudoranges for data fusion and avoids directly obtaining position information. Define the pseudorange between the receiver and satellite at time *t* as shown in Eq. (1):

1 $$ \rho_{a}^{e} (t) = R_{a}^{e} (t) + \delta_{a} (t) + \Omega^{e} + \zeta_{a}^{e} (t) $$

where $\rho_{a}^{e}$ is the observed pseudorange from receiver *a* to satellite *e*; $R_{a}^{e}$ is the true distance from receiver *a* to satellite *e*; $\delta_{a}$ is the clock error of receiver *a*; $\Omega^{e}$ is the common error related to satellite *e*, such as satellite clock error, atmospheric delay error, etc.; $\zeta_{a}^{e}$ is the non-common error related to satellite *e*, such as the thermal noise of receiver *a*, multipath interference and other non-line-of-sight errors.

When satellite *e* and satellite *j* are observed by receiver *a* and receiver *b* at the same time, the receiver clock error and satellite-related common error in Eq. (1) can be eliminated by the double differenced pseudoranges of the two satellites, as shown in Eq. (2):

2 $$ \begin{gathered} \rho_{ab}^{ej} (t)  = \rho_{a}^{e} (t) - \rho_{b}^{e} (t) - \rho_{a}^{j} (t) + \rho_{b}^{j} (t) \\ = R_{ab}^{ej} (t) + \zeta_{ab}^{ej} (t) \\ \end{gathered} $$

where $\rho_{ab}^{ej}$ represents the double differenced pseudorange observed by receiver *a* and receiver *b* on satellite *e* and satellite *j*; $R_{ab}^{ej}$ is the double differenced true distance from receiver *a* and receiver *b* to satellite *e* and satellite *j*; $\zeta_{ab}^{ej}$ is the non-common error residual of receiver *a* and receiver *b* to satellite *e* and satellite *j* that cannot be eliminated by double difference. Define the unit line-of-sight vector $\overrightarrow {{{{\varvec{\upkappa}}}_{sat} }}$ from receiver *rec* to satellite *sat*:

3 $$ \overrightarrow {{{{\varvec{\upkappa}}}_{sat} }} = \frac{{r_{sat} (t) - r_{rec} (t)}}{{\left\| {r_{sat} (t) - r_{rec} (t)} \right\|}} $$

where, $r_{sat}$ and $r_{rec}$ are satellite and receiver positions, respectively.

The single difference definition of the real distance between the receiver and the satellite is given by $\overrightarrow {{{{\varvec{\upkappa}}}_{sat} }}$:

4 $$ \left\{ {\begin{array}{*{20}l} {R_{a}^{e} - R_{b}^{e} = \overrightarrow {{{{\varvec{\upkappa}}}_{e} }}^{{\text{T}}} \overrightarrow {{{\mathbf{r}}_{ab} }} } \hfill \\ {R_{a}^{j} - R_{b}^{j} = \overrightarrow {{{{\varvec{\upkappa}}}_{j} }}^{{\text{T}}} \overrightarrow {{{\mathbf{r}}_{ab} }} } \hfill \\ \end{array} } \right. $$

where $\overrightarrow {{{{\varvec{\upkappa}}}_{e} }}$ and $\overrightarrow {{{{\varvec{\upkappa}}}_{j} }}$ are the unit line-of-sight vectors from receiver *a* (or receiver *b*) to satellite *e* and satellite *j*, respectively. $\overrightarrow {{{\mathbf{r}}_{ab} }}$ is the relative distance vector between receiver *a* and receiver *b*.

Then the double differenced true range $R_{ab}^{ej}$ can be obtained according to Eq. (4), as shown in Eq. (5):

5 $$ R_{ab}^{ej} (t) = \left[ {\overrightarrow {{{{\varvec{\upkappa}}}_{e} }} (t) - \overrightarrow {{{{\varvec{\upkappa}}}_{j} }} (t)} \right]^{{\text{T}}} \overrightarrow {{{\mathbf{r}}_{ab} }} (t) $$

Substituting Eq. (5) into Eq. (2), we can get:

6 $$ \rho_{ab}^{ej} (t) = \left[ {\overrightarrow {{{{\varvec{\upkappa}}}_{e} }} (t) - \overrightarrow {{{{\varvec{\upkappa}}}_{j} }} (t)} \right]^{{\text{T}}} \overrightarrow {{{\mathbf{r}}_{ab} }} (t) + \zeta_{ab}^{ej} (t) $$

It should be noted that since the positioning error of Global Positioning System (GPS) is generally within tens of meters, which is almost negligible compared with the distance from the navigation satellite to the earth's surface, the unit line-of-sight vector between the receiver and satellite can be obtained by using the approximate receiver position and the known satellite ephemeris^37^.

Doppler frequency shift is the signal frequency change caused by the relative movement between a satellite and a receiver, and can be used to obtain the moving speed of the receiver. In this paper, the definition of Doppler frequency shift $\vartheta_{a}^{e} (t)$ of satellite *e* by receiver *a* at time *t* is shown in Eq. (7):

7 $$ \vartheta_{a}^{e} (t) = \frac{qu}{c}\frac{{dR_{a}^{e} (t)}}{dt} + qu\frac{{d\delta_{a} (t)}}{dt} + qu\frac{{d\Omega^{e} (t)}}{dt} + \gamma_{a}^{e} (t) $$

where *c* is the speed of light; *qu* is the carrier frequency of the satellite signal; $\gamma_{a}^{e}$ is the Doppler observation error. The double differenced Doppler frequency shift $\vartheta_{ab}^{ej} (t)$ is obtained as:

8 $$ \vartheta_{ab}^{ej} (t) = \frac{qu}{c}\frac{{dR_{ab}^{ej} (t)}}{dt} + \gamma_{ab}^{ej} (t) $$

in Eq. (8), $\gamma_{ab}^{ej}$ is the residual error of the Doppler observation error between satellite *e* and satellite *j* by receiver *a* and receiver *b*. Substituting the double differenced true distance into it gets:

9 $$ \vartheta_{ab}^{ej} (t) = \frac{qu}{c}\left[ {\overrightarrow {{{{\varvec{\upkappa}}}_{e} }} (t) - \overrightarrow {{{{\varvec{\upkappa}}}_{j} }} (t)} \right]^{{\text{T}}} \overrightarrow {{{\mathbf{v}}_{ab} }} (t) + \gamma_{ab}^{ej} (t) $$

where $\overrightarrow {{{\mathbf{v}}_{ab} }}$ is the relative velocity between receiver *a* and receiver *b*.

### UWB observation

UWB takes the half of the product of signal propagation time and propagation speed as the relative distance, and uses at least three reference stations with known coordinates to obtain the three-dimensional coordinates of the target point through resection. Under normal circumstances, the vehicles run on the same road and the driving track is regular, this paper assumes that there are no obstacles between UWB transceivers. In Shen's work^7^, the quadratic polynomial error model is derived using the real pulse UWB data of multispectral Solutions Inc. (MSSI) provided by Joseph et al^38^. The experimental results by Shenshowed that the probability density of the UWB ranging error can be assumed to obey the zero-mean Gaussian distribution with variance of 0.3 ${\text{m}}^{{2}}$, and the UWB measurement data were simulated through these conclusions. In order to control variables and more closely compare and analyze the performance of the proposed algorithm, the UWB data used in this paper remains the same as that inShen’s work. The definition of UWB ranging here meets the following requirements:

10 $$ \hat{R}_{ab} = \sqrt {(x_{a} - x_{b} )^{{2}} + (y_{a} - y_{b} )^{{2}} + (s_{a} - s_{b} )^{{2}} } + \varsigma_{ab} $$

where $x$, $y$, $s$ are the three-dimensional positions of vehicles *a* and *b*, respectively; $\varsigma_{ab}$ is the observation noise at UWB distance.

### System modeling

The state equation of the cooperative navigation system is defined as:

11 $$ {{\varvec{\upchi}}}(t + \tau ) = {\mathbf{\Phi \chi }}(t) + {{\varvec{\Gamma}}}\varsigma (t) $$

where $\tau$ is the measurement interval; ${{\varvec{\upchi}}}$ is the state vector; ${{\varvec{\Phi}}}$ is the state transition matrix; ${{\varvec{\Gamma}}}$ is the process noise model; $\varsigma$ is the three-axis relative acceleration noise, and satisfies a zero-mean Gaussian distribution with a standard deviation of $\sigma = {1}$. The covariance matrix of the process noise is set to ${\mathbf{Q}} = \sigma^{{2}} {\mathbf{\Gamma \Gamma }}^{{\text{T}}}$. The specific representations of each matrix are as follows:

$${{\varvec{\upchi}}} = \left[ {\begin{array}{*{20}l} {\overrightarrow {{{\mathbf{r}}_{ab} }} } \hfill & {\overrightarrow {{{{\varvec{\upnu}}}_{ab} }} } \hfill \\ \end{array} } \right]^{{\text{T}}}, {{\varvec{\Phi}}} = \left[ {\begin{array}{*{20}c} {{\mathbf{I}}_{{3}} } & {\tau {\mathbf{I}}_{{3}} } \\ {{\mathbf{0}}_{{3}} } & {{\mathbf{I}}_{{3}} } \\ \end{array} } \right], {{\varvec{\Gamma}}} = \left[ {\begin{array}{*{20}l} {{0}{\text{.5}}\tau^{{2}} {\mathbf{I}}_{{3}} } \hfill & {\tau {\mathbf{I}}_{{3}} } \hfill \\ \end{array} } \right]^{{\text{T}}}$$

The measurement equation of the system is showed as the Eq. (12):

12 $$ {\mathbf{Z}}(t) = h({{\varvec{\upchi}}}(t)) + {{\varvec{\upzeta}}}(t) $$

where ${\mathbf{Z}}$ is the vector of measurements, including the double differenced pseudoranges, double differenced Doppler frequency shifts and relative distances between vehicles; $h$ is a nonlinear function composed of Eqs. (6), (9) and (10); ${{\varvec{\upzeta}}}$ is the noise of the observation vector; the covariance matrix of the system's observations is defined as ${{\varvec{\Pi}}}$. If the number of simultaneously observed GPS satellites is L, the system observation vector and its observation error can be defined as:

$$ {\mathbf{Z}} = \left[ {\begin{array}{*{20}c} {\rho_{ab}^{{{12}}} } & \cdots & {\rho_{ab}^{{{\text{1L}}}} } & {\vartheta_{ab}^{{{12}}} } & \cdots & {\vartheta_{ab}^{{{\text{1L}}}} } & {\hat{R}_{ab} } \\ \end{array} } \right]^{{\text{T}}} $$

$$ {{\varvec{\upzeta}}} = \left[ {\begin{array}{*{20}l} {\zeta_{ab}^{{{12}}} } \hfill & \cdots \hfill & {\zeta_{ab}^{{{\text{1L}}}} } \hfill & {\gamma_{ab}^{{{12}}} } \hfill & \cdots \hfill & {\gamma_{ab}^{{{\text{1L}}}} } \hfill & {\varsigma_{ab} } \hfill \\ \end{array} } \right]^{{\text{T}}} $$

In order to obtain the covariance matrix of observation noise, we first need to define the observation error variance of pseudo range $\sigma_{\rho }^{{2}}$, Doppler frequency shift $\sigma_{\vartheta }^{{2}}$ , UWB ranging error $\sigma_{r}^{{2}}$ and the matrix A as the follow:

13 $$ {\mathbf{A}} = \left[ {\begin{array}{*{20}l} {{\mathbf{1}}_{{({\text{L}} - {1}) \times {1}}} } \hfill & { - {\mathbf{I}}_{{({\text{L}} - {1})}} } \hfill & { - {\mathbf{1}}_{{({\text{L}} - {1}) \times {1}}} } \hfill & {{\mathbf{I}}_{{({\text{L}} - {1})}} } \hfill \\ \end{array} } \right] $$

where ${\mathbf{1}}$ represents a matrix whose elements are all 1. Then, the observation noise matrix can be obtained, as shown in Eq. (14):

14 $$ {{\varvec{\Pi}}} = \left[ {\begin{array}{*{20}c} {{{\varvec{\Pi}}}_{\rho } } & {{\mathbf{0}}_{{{\text{L}} - {1}}} } & {\mathbf{0}} \\ {{\mathbf{0}}_{{{\text{L}} - {1}}} } & {{{\varvec{\Pi}}}_{\vartheta } } & {\mathbf{0}} \\ {{\mathbf{0}}_{{{1} \times ({\text{L}} - {1})}} } & {{\mathbf{0}}_{{{1} \times ({\text{L}} - {1})}} } & {{{\varvec{\Pi}}}_{r} } \\ \end{array} } \right] $$

where $${{\varvec{\Pi}}}_{\rho } = \sigma_{\rho }^{{2}} {\mathbf{AA}}^{{\text{T}}}$$, $${{\varvec{\Pi}}}_{\vartheta } = \sigma_{\vartheta }^{{2}} {\mathbf{AA}}^{{\text{T}}}$$, $${{\varvec{\Pi}}}_{r} = \sigma_{r}^{{2}}$$.

## Robust cubature fission particle filter

### Classical particle filter

Filtering can fuse a variety of measurement data, and use these data to finally obtain the estimated target parameters. Its purpose is to estimate, but the process can be regarded as the fusion of multiple measurement data. The particle filter uses weighted particles to perform Bayesian estimation based on Monte Carlo idea. Under the assumption of the first-order Markov process, the algorithm logic is divided into two stages, namely state prediction and time update^39^. In the state prediction stage, the algorithm predicts the particle state through the kinematic model of the system. Liu et al. have proved that $q\left( {{{\varvec{\upchi}}}_{k} |{{\varvec{\upchi}}}_{{{0:}k - {1}}} ,{\mathbf{Z}}_{{{1:}k}} } \right)$ is the optimal approximation to a posteriori distribution $p\left( {{{\varvec{\upchi}}}_{k} |{\mathbf{Z}}_{{{1:}k}} } \right)$ ^40^. However, in the actual algorithm implementation, because it is difficult to realize, a suboptimal approximation $q\left( {{{\varvec{\upchi}}}_{k} |{\mathbf{Z}}_{k} } \right)$ (IDF) is often used to randomly extract the particle ${{\varvec{\upchi}}}_{k}^{i}$ to generate new particle states, and each state is evaluated by the particle weight obtained by time update, as shown in the following equation:

15 $$ \begin{gathered} p\left( {{{\varvec{\upchi}}}_{k} |{\mathbf{Z}}_{{{1:}k}} } \right) = q\left( {{{\varvec{\upchi}}}_{k} |{\mathbf{Z}}_{k} } \right) \\ = \sum\limits_{{i = {1}}}^{N} {\tilde{w}_{k}^{i} } \delta ({{\varvec{\upchi}}}_{k} - {{\varvec{\upchi}}}_{k}^{i} ) \\ \end{gathered} $$

where *N* is the number of particles and $\delta ()$ is the Dirac function. The particle weight is calculated as follows:

16 $$ \tilde{w}_{k}^{i} = \tilde{w}_{{k - {1}}}^{i} \frac{{p\left( {{\mathbf{Z}}_{k} |{{\varvec{\upchi}}}_{k}^{i} } \right)p({{\varvec{\upchi}}}_{k}^{i} |{{\varvec{\upchi}}}_{{k - {1}}}^{i} )}}{{q({{\varvec{\upchi}}}_{k}^{i} |{{\varvec{\upchi}}}_{{k - {1}}}^{i} ,{\mathbf{Z}}_{k} )}} $$

where $p\left( {{\mathbf{Z}}_{k} |{{\varvec{\upchi}}}_{k}^{i} } \right)$ is the likelihood function, $p({{\varvec{\upchi}}}_{k}^{i} |{{\varvec{\upchi}}}_{{k - {1}}}^{i} )$ is the state transition probability, and $q({{\varvec{\upchi}}}_{k}^{i} |{{\varvec{\upchi}}}_{{k - {1}}}^{i} ,{\mathbf{Z}}_{k} )$ is the importance density. In order to update the particle state, the weight needs to be normalized:

17 $$ \tilde{w}_{k}^{i} = \tilde{w}_{k}^{i} /\sum\limits_{{i = {1}}}^{N} {\tilde{w}_{k}^{i} } $$

Considering the problem of particle degradation, resampling technology is used in classical particle filters. First, the effective particle number $N_{eff}$ needs to be judged. When the number of effective particles is less than the specified threshold $N_{th}$, the algorithm performs resampling to avoid resource waste and improve computing efficiency by eliminating small-weight particles. The calculation of effective particle number is as follows:

18 $$ N_{eff} = \frac{{1}}{{\sum\limits_{{i = {1}}}^{N} {(\tilde{w}_{k}^{i} )^{{2}} } }} $$

The corresponding threshold $N_{th}$ should be set according to engineering experience, generally 0.3 ~ 0.8 $N_{eff}$. Resampling is performed if $N_{eff}$ is less than the threshold. Otherwise, the posterior estimation of the state is obtained directly to complete the particle filter.

### Importance density function design

As mentioned earlier, IDF has a crucial impact on the performance of a particle filter. Li et al. has proved that the measurement with significant noise will produce a less precise PD when introduced into the IDF, causing interference to the simulation of a posteriori distribution^41^. For further explanation, we take CKF as IDF as an example. CKF is an L2 norm estimation based on Gaussian distribution. Although the cubature law compensates some errors from the perspective of system nonlinear error in the generation stage of PD. The L2 idea of minimum variance will square the error in the calculation process and amplify the accuracy loss caused by outliers. Therefore, the measurement outliers far away from the sample population have an obvious impact on the residuals squares sum of the sample population, resulting in the decline or even divergence of the overall estimation accuracy of CPF. Different from L2 estimation, the error probability distribution of L1 estimation belongs to Laplace distribution, which has more significant "thick tail" characteristics^42–44^, when the variance of Laplace distribution is equal to that of Gaussian distribution, Laplace distribution has a higher probability density near and far from the mean than Gaussian distribution. So it is more suitable for the likelihood description of significant noise. At the same time, the error growth rate of L1 norm estimation is "linear" and significantly slower than the quadratic growth of L2 norm error, which makes L1 estimation have better suppression effect and stronger robustness against colored noise and outliers.

In this paper, we first fuse L2 and L1 estimation based on Huber function in the more advanced CKF algorithm for nonlinear systems, and combine Gaussian distribution and Laplace distribution to better approximate the posterior probability of the system under the cubature law; secondly, the measurement update of CKF is transformed into linear regression, and solved by Huber M estimation. The M estimation uses Huber weight function as its weight function, which can give full play to the robustness of L1. So the algorithm will reduce the weight of the disturbed measurement, truncate and average the filtering innovation to achieve robustness. 

#### Cubature point calculation

CKF approximates the probability density function by weighting the cubature points. In order to realize function transfer, the algorithm first needs to generate a cubature point set with equal weight according to the cubature criterion:

19 $$ \left\{ {\begin{array}{*{20}l} {\xi_{i} = \sqrt n [eye]_{i} ,i = {1,}...{,2}n} \hfill \\ {\omega_{i} = \frac{{1}}{{{2}n}}} \hfill \\ \end{array} } \right. $$

where $\xi_{i}$ is the cubature point; *n* is the state dimension; $[eye]_{i}$ represents the projection coordinates of the unit vector in the *n*-dimensional space on each axis; $\omega_{i}$ is the weight of a cubature point.

#### State update

First, the cubature point ${{\varvec{\upxi}}}_{i}$ is used to obtain the updated state cubature point ${{\varvec{\upchi}}}_{{i,k - {1|}k - {1}}}$ according to the posterior state estimation $\widehat{{{\varvec{\upchi}}}}_{{k - {1|}k - {1}}}$ and its covariance matrix ${\mathbf{P}}_{{k - {1|}k - {1}}}$ at time $k - {1}$:

20 $$ {{\varvec{\upchi}}}_{{i,k - {1|}k - {1}}} = \sqrt {{\mathbf{P}}_{{k - {1|}k - {1}}} } {{\varvec{\upxi}}}_{i} + \widehat{{{\varvec{\upchi}}}}_{{k - {1|}k - {1}}} $$

Second, based on the system dynamics model, the function is transferred through the state cubature points, and the approximation ${{\varvec{\upchi}}}_{{i,k{|}k - {1}}}^{*}$ of the state transition probability density of each state cubature point is obtained:

21 $$ {{\varvec{\upchi}}}_{{i,k{|}k - {1}}}^{*} = f\left( {{{\varvec{\upchi}}}_{{i,k - {1|}k - {1}}} } \right) $$

Finally, the system priori state is obtained by equal-weighted fusion of the state cubature point information, and the priori error covariance matrix is estimated:

22 $$ \widehat{{{\varvec{\upchi}}}}_{{k|k - {1}}} = \frac{{1}}{{{2}n}}\sum\limits_{{i = {1}}}^{{{2}n}} {{{\varvec{\upchi}}}_{{i,k|k - {1}}}^{*} } $$

23 $$ {\mathbf{P}}_{{k|k - {1}}} = \frac{{1}}{{{2}n}}\sum\limits_{{i = {1}}}^{{{2}n}} {{{\varvec{\upchi}}}_{{i,k|k - {1}}}^{*} } {{\varvec{\upchi}}}_{{i,k|k - {1}}}^{{*{\text{T}}}} - \widehat{{{\varvec{\upchi}}}}_{{k|k - {1}}} \widehat{{{\varvec{\upchi}}}}_{{k|k - {1}}}^{{\text{T}}} + {\mathbf{Q}}_{{k - {1}}} $$

where $\widehat{{{\varvec{\upchi}}}}_{{k|k - {1}}}$ and ${\mathbf{P}}_{{k|k - {1}}}$ are the priori state and its covariance matrix after the weighted average of each cubature point at time $k$.

#### Measurement update

Measurement update needs to use $\widehat{{{\varvec{\upchi}}}}_{{k|k - {1}}}$ and ${\mathbf{P}}_{{k|k - {1}}}$ obtained from state update. First, calculate the new cubature point ${{\varvec{\upchi}}}_{{i,k|k - {1}}}$ according to the a priori error covariance matrix:

24 $$ {{\varvec{\upchi}}}_{{i,k|k - {1}}} = \sqrt {{\mathbf{P}}_{{k|k - {1}}} } {{\varvec{\upxi}}}_{i} + \widehat{{{\varvec{\upchi}}}}_{{k|k - {1}}} $$

In order to obtain the measurement likelihood description of each cubature point, it is necessary to use the new cubature point ${{\varvec{\upchi}}}_{{i,k|k - {1}}}$ to generate the observation cubature point $z_{{i,k|k - {1}}}$ through the observation equation $h$:

25 $$ z_{{i,k|k - {1}}} = h\left( {{{\varvec{\upchi}}}_{{i,k|k - {1}}} } \right) $$

Second, perform equal-weighted fusion estimation on the measured cubature points to obtain the measured estimated value $\hat{z}_{{k|k - {1}}}$:

26 $$ \hat{z}_{{k|k - {1}}} = \frac{{1}}{{{2}n}}\sum\limits_{{i = {1}}}^{{{2}n}} {z_{{i,k|k - {1}}} } $$

Finally, the measurement variance matrix ${\mathbf{P}}_{{zz,k|k - {1}}}$ and the covariance matrix between state and measurement ${\mathbf{P}}_{{\chi z,k|k - {1}}}$ between the state prediction and the measurement prediction are obtained. The specific equations are as follows:

27 $$ {\mathbf{P}}_{{zz,k|k - {1}}} = \frac{{1}}{{{2}n}}\sum\limits_{{i = {1}}}^{{{2}n}} {z_{{i,k|k - {1}}} } z_{{i,k|k - {1}}}^{{\text{T}}} - \widehat{z}_{{k|k - {1}}} \widehat{z}_{{k|k - {1}}}^{{\text{T}}} + {{\varvec{\Pi}}} $$

28 $$ {\mathbf{P}}_{{\chi z,k|k - {1}}} = \sum\limits_{{i = {1}}}^{{{2}n}} {\omega_{i} } {{\varvec{\upchi}}}_{{i,k|k - {1}}} z_{{i,k|k - {1}}}^{{\text{T}}} - \widehat{{{\varvec{\upchi}}}}_{{k|k - {1}}} \widehat{z}_{{k|k - {1}}}^{{\text{T}}} $$

#### M estimation to solve linear regression

After the measurement update is completed, a linear regression equation needs to be constructed, and the measurement update is further transformed into solving the linear regression problem.

Using state prediction and measurements combined with observation equation to construct linear regression equation. The relationship between true state and valuation can be expressed as:

29 $$ \delta {{\varvec{\upchi}}}_{k} { = }{{\varvec{\upchi}}}_{k} - \widehat{{{\varvec{\upchi}}}}_{{k|k - {1}}} $$

where ${{\varvec{\upchi}}}_{k}$ is the true value of the state at time $k$, and $\delta {{\varvec{\upchi}}}_{k}$ is the state prediction error.

The measurement Eq. (12) can be approximated as:

30 $$ {\mathbf{Z}}_{k} { = }h\left( {\widehat{{{\varvec{\upchi}}}}_{{k|k - {1}}} } \right) + {\mathbf{H}}_{k} ({{\varvec{\upchi}}}_{k} - \widehat{{{\varvec{\upchi}}}}_{{k|k - {1}}} ) + {{\varvec{\upzeta}}}_{k} $$

where ${\mathbf{H}}_{k} = \left[ {\left( {{\mathbf{P}}_{{k|k - {1}}} } \right)^{{ - 1}} {\mathbf{P}}_{{\chi z,k|k - {1}}} } \right]^{{\text{T}}}$. Combining Eq. (29) and Eq. (30) to obtain a linear regression equation:

31 $$ \left[ {\begin{array}{*{20}c} {{\mathbf{Z}}_{k} - h\left( {\widehat{{{\varvec{\upchi}}}}_{{k|k - {1}}} } \right) + {\mathbf{H}}_{k} \widehat{{{\varvec{\upchi}}}}_{{k|k - {1}}} } \\ {\widehat{{{\varvec{\upchi}}}}_{{k|k - {1}}} } \\ \end{array} } \right] = \left[ {\begin{array}{*{20}c} {{\mathbf{H}}_{k} } \\ {\mathbf{I}} \\ \end{array} } \right]{{\varvec{\upchi}}}_{k} + \left[ {\begin{array}{*{20}c} {{{\varvec{\upzeta}}}_{k} } \\ { - \delta {{\varvec{\upchi}}}_{k} } \\ \end{array} } \right] $$

where ${\mathbf{I}}$ is the unit matrix. Simplify Eq. (31) as follows:

32 $$ {{\varvec{\Xi}}}_{k} = {{\varvec{\Theta}}}_{k} {{\varvec{\upchi}}}_{k} + {{\varvec{\Lambda}}} $$

Each matrix variable is specifically defined as:

33 $$ {{\varvec{\Xi}}}_{k} = {\mathbf{T}}_{k}^{{ - 1/2}} \left[ {\begin{array}{*{20}c} {{\mathbf{Z}}_{k} - h\left( {\widehat{{{\varvec{\upchi}}}}_{{k|k - {1}}} } \right) + {\mathbf{H}}_{k} \widehat{{{\varvec{\upchi}}}}_{{k|k - {1}}} } \\ {\widehat{{{\varvec{\upchi}}}}_{{k|k - {1}}} } \\ \end{array} } \right] $$

34 $$ {{\varvec{\Theta}}}_{k} = {\mathbf{T}}_{k}^{{ - 1/2}} \left[ {\begin{array}{*{20}c} {{\mathbf{H}}_{k} } \\ {\mathbf{I}} \\ \end{array} } \right] $$

35 $$ {{\varvec{\Lambda}}}_{k} = {\mathbf{T}}_{k}^{{ - 1/2}} \left[ {\begin{array}{*{20}c} {{{\varvec{\upzeta}}}_{k} } \\ { - \delta {{\varvec{\upchi}}}_{k} } \\ \end{array} } \right] $$

where $${\mathbf{T}}_{k} = diag([{{\varvec{\Pi}}}_{k}  {\mathbf{P}}_{{k|k - {1}}} ])$$.

Huber M estimation is used to solve the above linear regression. Define loss function:

36 $$ J\left( {{{\varvec{\upchi}}}_{k} } \right) = \sum\limits_{{i = {1}}}^{M} \rho \left( {\eta_{i} } \right) $$

where *M* is the total dimension of the state and measurements, and $\eta_{i}$ is the i-th value of residual vector ${{\varvec{\upeta}}} = {{\varvec{\Theta}}}_{k} {{\varvec{\upchi}}}_{k} - {{\varvec{\Xi}}}_{k}$.

The Huber function as Function $\rho \left( {\eta_{i} } \right)$, shown as follows:

37 $$\rho \left( {\eta_{i} } \right) = \left\{ {\begin{array}{*{20}l} {\frac{{1}}{{2}}\eta_{i}^{{2}} ,\left| {\eta_{i} } \right| \ge \gamma } \hfill \\ {\gamma \left| {\eta_{i} } \right| - \frac{{1}}{{2}}\gamma^{{2}} ,\left| {\eta_{i} } \right| > \gamma } \hfill \\ \end{array} } \right. $$

where $\gamma$ is the constraint factor, in this paper, it is set to 0.25. When $\gamma$ is close to 0, the $\rho$ function is close to the Laplace distribution of L1 norm estimation. When $\gamma$ is close to infinity, the $\rho$ function is close to the Gaussian distribution of L2 norm estimation. M estimation requires the minimum loss function, i.e., it is equivalent to

38 $$ \sum\limits_{{i = {1}}}^{M} \varphi \left( {\eta_{i} } \right)\frac{{\partial \eta_{i} }}{\partial \chi } = {0} $$

where

39 $$  \varphi \left( {\eta_{i} } \right) = \rho {^{\prime}}\left( {\eta_{i} } \right){ = }\left\{ {\begin{array}{*{20}l} {\eta_{i} ,} \hfill & {\left| {\eta_{i} } \right| \ge \gamma } \hfill \\ {\gamma {\text{sgn}} \left( {\eta_{i} } \right),} \hfill & {\left| {\eta_{i} } \right| > \gamma } \hfill \\ \end{array} } \right. $$

Defining $\psi \left( {\eta_{i} } \right) = \varphi \left( {\eta_{i} } \right)/\eta_{i}$, and the matrix ${{\varvec{\Psi}}}$ can be obtained as follows:

40 $$ {{\varvec{\Psi}}} = diag\left[ {\psi \left( {\eta_{i} } \right)} \right] $$

Using the above-mentioned matrices to simplify Eq. (38), we obtain Eq. (41):

41 $$ {{\varvec{\Theta}}}_{k}^{{\text{T}}} {{\varvec{\Psi}}}\left( {{{\varvec{\Theta}}}_{k} {{\varvec{\upchi}}}_{k} - {{\varvec{\Xi}}}_{k} } \right) = {0} $$

Defining $\mu$ as the number of iterations, using the iterative method to solve Eq. (41), a posteriori state ${\hat{\mathbf{\chi }}}_{k}$ at time $k$ can be obtained:

42 $$ {\hat{\mathbf{\chi }}}_{k}^{{(\mu + {1})}} = \left( {{{\varvec{\Theta}}}_{k}^{{\text{T}}} {{\varvec{\Psi}}}^{\mu } {{\varvec{\Theta}}}_{k} } \right)^{{ - 1}} {{\varvec{\Theta}}}_{k}^{{\text{T}}} {{\varvec{\Psi}}}^{\mu } {{\varvec{\Xi}}}_{k} $$

The corresponding a posteriori error covariance matrix ${\mathbf{P}}_{k|k}$ is calculated as follows:

43 $$ {\mathbf{P}}_{k|k} = \left( {{{\varvec{\Theta}}}_{k}^{{\text{T}}} {\mathbf{\Psi \Theta }}_{k} } \right)^{{ - {1}}} $$

### Weight reconstruction

Resampling is a method to eliminate the low weight particles to avoid the waste of computing resources caused by particle degradation. At the same time, by selecting and copying the high weight particles, resampling can maintain the confidence level of the particle swarm while keeping the total number of particles unchanged. However, this will destroy the diversity of particles. After several iterations, the algorithm will approximate the posterior distribution by only a few high weight particles in the initial particle swarm. The most extreme case is that the offspring particles are the offspring of a "most reliable" particle in the primary generation, i.e., the sample impoverishment. The simplest way to avoid sample impoverishment is to increase the number of particles, but this method is not efficient and will greatly increase the computational load.

In order to further alleviate the sample impoverishment and minimize the increased computational load, fission technology is adopted to derive particles before resampling^45^. First, when the number of effective particles is less than the threshold, the particle swarm is arranged in the order of weight from small to large. Second, fission: take the current particle as the parent, select the first $N_{th}$ high weight particles, take their state as the mean of Gaussian distribution, randomly select the state particles from this distribution to obtain the offspring, and use the offspring particles to replace the low weight particles in the parent in turn,the number of fission particles is proportional to the weight of the parent particles . Finally, normalize the weight of the new particle swarm, take each parent and its offspring particles as a group, evenly distribute the weight of the parent particle, and complete the weight reconstruction of the particle swarm. Compared with other methods of weight reconstruction, the fission method is simple to calculate and easy to implement, which can further alleviate the problem of sample impoverishment.

## Vehicle test results and analysis

**Figure 1:**
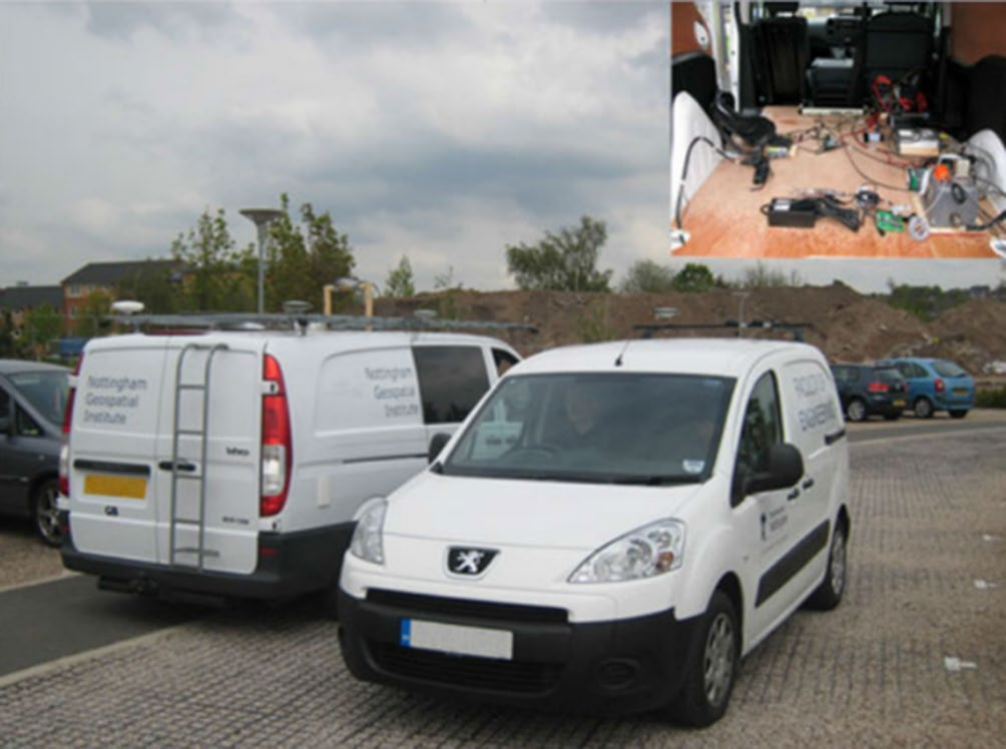
Test environment and experimental settings.

In order to verify the performance of the RCFPF algorithm, compare and analyze its impact on the relative position perception of cooperative navigation, the data used in the experiment were consistent with that in the experiment by Shen^7^. In order to obtain the original data of the algorithm, the relative distance between vehicles was obtained by UWB based on MSSI. Meanwhile, in order to obtain the reference truth value, the Leica GS10 receiver and the Novatel INS-LCI system integrating GNSS-INS were equipped on vehicles *a* and *b*, respectively, and the real time kinematic (RTK) positioning of them was implemented to obtained high-precision position estimations, which was used as the truth value to evaluate the algorithm performance. The pseudorange, Doppler frequency shift obtained by satellite receiver and UWB range were used to directly execute the proposed algorithm. Figure 1 shows the test environment and experimental settings. Table 1 shows the accuracy of the equipment under RTK.

**Table 1:** RTK position accuracy parameters.

Equipment	Horizontal accuracy (m)	Vertical accuracy (m)
Leica GS10	0.01	0.02
Novatel INS-LCI	0.02	0.05

The data time in the experiment was about 9 min. During this time, the two vehicles transmitted measurement information through UWB communication. The data sampling rate of GPS was 1 Hz. At the same time, the Differential Global Position System (DGPS) correction was executed through the GPS observations by a reference station of Nottingham Institute of geospatial research. In order to ensure a wide field of vision in the sky during data acquisition and ensure that the receiver can observe as many satellites as possible, the acquisition location is Clifton Boulevard between Derby road and Loughborough Road Road, Nottingham, UK. In the experiment, the initial relative position of the two vehicles is determined by GPS single point positioning, and the initial relative speed is set to zero. The initial state covariance matrix is set to ${100}{ \times }{\mathbf{I}}_{{6}}$, meanwhile $\sigma_{\rho }^{{2}} = \sigma_{\vartheta }^{{2}} = {1}$ which can be used to get the measurement noise matrix ${{\varvec{\Pi}}}$ according to the Eq. (14). The experiment is based on the EKF, CPF, STPF and RCFPF to perform data fusion and analysis to draw our conclusions.

**Figure 2:**
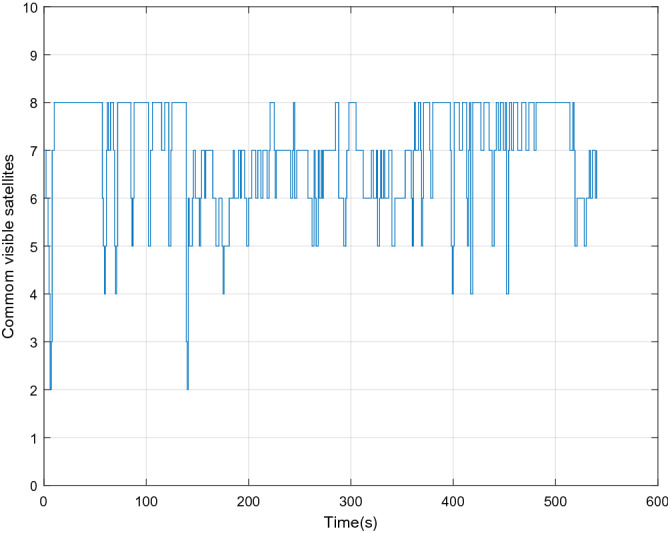
Number of commonly visible satellites.

**Figure 3:**
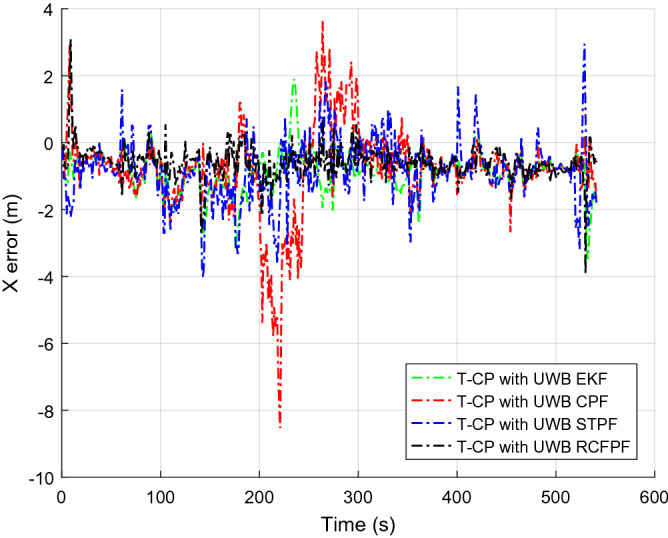
X-axis distance error.

**Figure 4:**
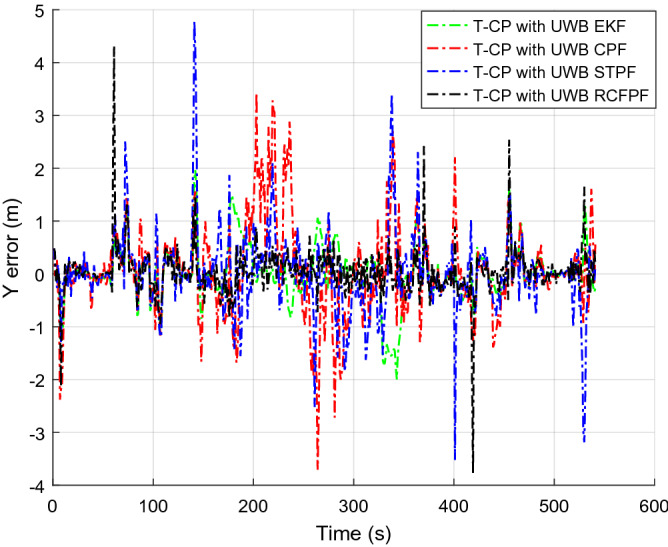
Y-axis distance error.

**Figure 5:**
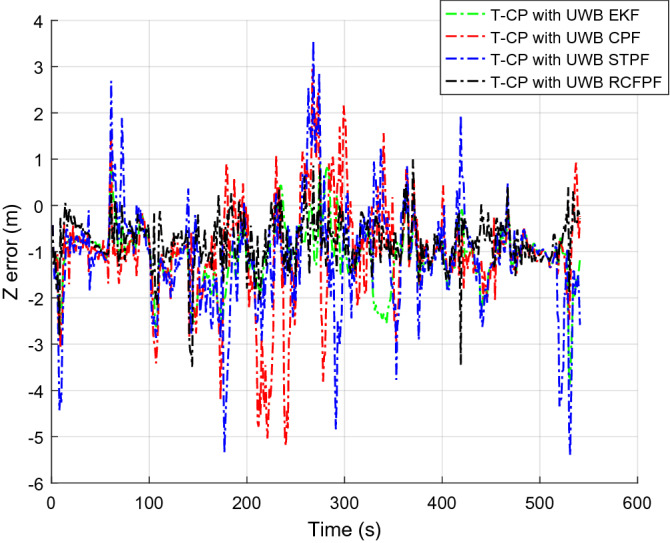
Z-axis distance error.

**Figure 6:**
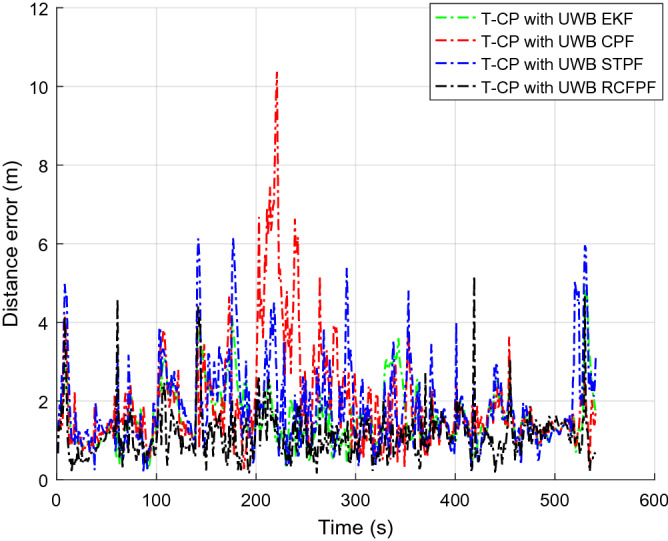
Three-dimensional distance error.

Figure 2 shows the number of visible satellites at the time of data collection. It can be seen from the figure that the number of commonly visible satellites is greater than the four required for positioning in most of the time during the data acquisition. In a small amount of time less than four, we set the innovation in the Kalman filter to zero, enlarged the corresponding observation covariance matrix, and increased the distrust of the measurement to ensure the normal update of the state estimation. In order to highlight the accuracy of the approximation of a posterior distribution by RCFPF, the algorithm used a smaller number of particles and set it to 60. Because for approximate efficiency, it is meaningless to use a large number of particles for verification. And the EKF as the evaluation standard of particle approximation accuracy to judge whether these particles have accurately approximated the posterior probability from the accuracy of estimation results. The tight combination with UWB is defined as "T-CP with UWB". Figures 3, 4, 5 and 6 show the coordinate estimation errors of four "T-CP with UWB" filtering algorithms for three-axis and three-dimensional population.

As shown in Figs. 3, 4, 5 and 6, the accuracy of CPF, STPF, EKF, and RCFPF are successively improved, and the accuracy of RCFPF in each axis direction and overall accuracy is the best. This is because in the case of fewer particles, CPF and STPF used one-sided IDF, the PD that they produced cannot well approximate the posteriori distribution, which led to the fact that the accuracy of the two is inferior to that of EKF, and because STPF has adaptive function, its accuracy was slightly better than that of CPF. RCFPF combines the Gaussian distribution and Laplace distribution to better approximate the system probability density, and combines the L1 norm to suppress observation noise, increasing the number of particles in the likelihood region, so it can obtain more accurate results under the condition of fewer particles. At the same time, it can be found that CPF has error fluctuation around 0 s and 200 s, which is because the DOP at these time is poor. Compared with other algorithms at this time, CPF is the most sensitive to DOP. The overall fluctuation of STPF is the strongest. This is because the state equation of the system is a linear equation and there is model error. STPF has a certain adaptive function. It mistakenly transfers the model error to the measurement error for adjustment, resulting in excessive adjustment at some times and large fluctuation of the results. Therefore, STPF may not be suitable for complex integrated navigation with model errors compared with other algorithms.

**Figure 7:**
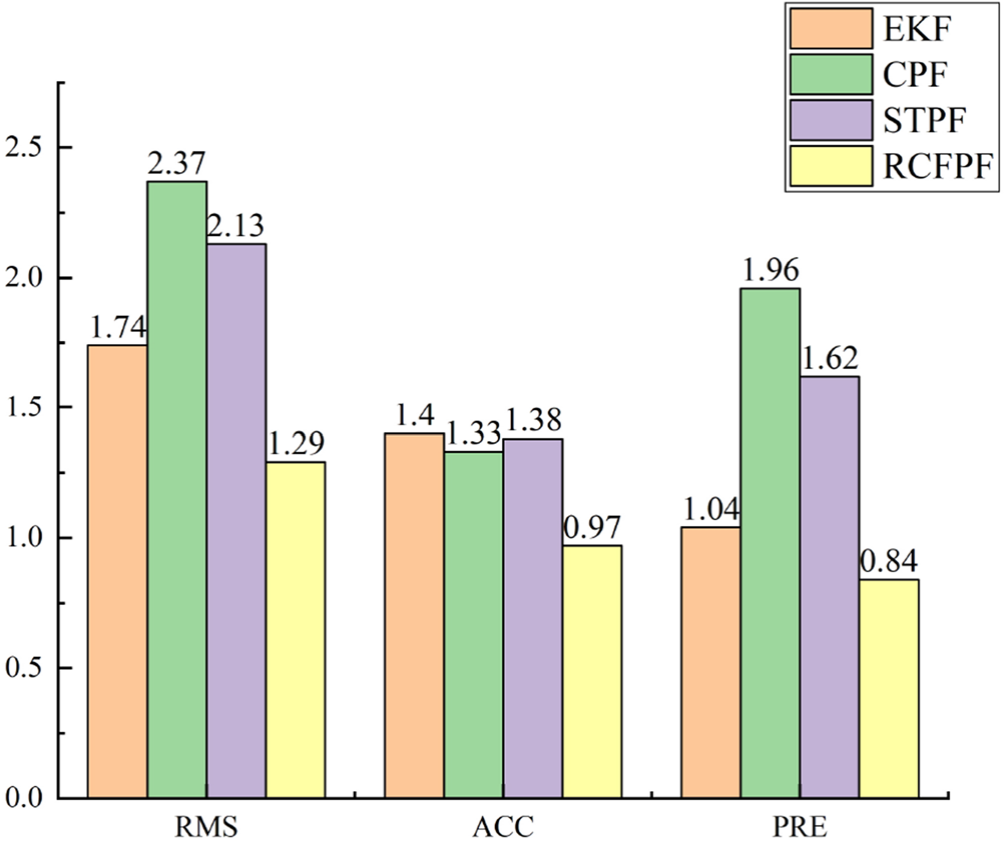
Algorithm quantization standards.

In order to compare the performance of four data fusion algorithms in more detail, and simultaneously prove the robustness and accuracy of RCFPF. According to the filter estimation error $\left[ {{\vec{\mathbf{r}}^{\prime}}(t) - {\vec{\mathbf{r}}}(t)} \right]$ and its covariance matrix ${{\varvec{\upalpha}}} = {\text{cov}} \left[ {{\vec{\mathbf{r}}^{\prime}}(t) - {\vec{\mathbf{r}}}(t)} \right]$, the accuracy $ACC = abs(\ell^{{ - 1}} \sum\nolimits_{{t = {1}}}^{\ell } {\left[ {{\vec{\mathbf{r}}^{\prime}}(t) - {\vec{\mathbf{r}}}(t)} \right]} )$ representing the relative position estimation deviation and the precision $PRE = sqrt(\sum\nolimits_{{i = {1}}}^{{3}} {{{\varvec{\upalpha}}}_{ii} } )$ representing the algorithm robustness are defined outside root mean squared (RMS). Where ${\vec{\mathbf{r}}^{\prime}}$ is the relative position obtained by executing the algorithm; ${\vec{\mathbf{r}}}$ is the true relative position obtained by RTK; $\ell$ is the total number of measurement epochs; $\alpha_{ii}$ is the *i*th diagonal element of the covariance matrix ${{\varvec{\upalpha}}}$. The quantization standards of the four algorithms are shown in Fig. 7:

In order to further express the improvement of algorithm $\varpi$ relative to algorithm $\varepsilon$, the variable $\beta$ is defined as follows:

44 $$ \beta = \left[ {\frac{{{\text{Standard}} \, \varepsilon {\text{ - Standard}} \, \varpi }}{{{\text{Standard}} \, \varepsilon }}} \right] \times {100}\% $$

Utilizing $\beta$ to quantitatively compare the RMS, *ACC* and *PRE* of the four algorithms, the percentage of RCFPF improvement over EKF, CPF and STPF in the three standards is obtained as follows.

It can be seen from Table 2 the RMS, accuracy, and robustness of RCFPF have greatly improved compared to the other three algorithms. The experimental results have confirmed that the algorithm can further optimize PD by improving the probability approximation method and suppressing the observation noise, which improves the estimation performance of particle filter in multi-source cooperative navigation and provides a new system quality control method for vehicle cooperative navigation.

**Table 2:** Experimental results and performance improvement.

Methods	RMS (m)	Accuracy (m)	Precision (m)
RCFPF over EKF	26.12%	30.67%	18.46%
RCFPF over CPF	45.68%	27.22%	56.85%
RCFPF over STPF	39.56%	29.76%	47.85%

## Conclusion

Based on cooperative navigation as a platform, this paper proposes a robust cubature fission particle filter for multi-source data fusion. The algorithm can fit the a posteriori probability density of the system more flexibly through the Gaussian distribution and Laplace distribution. At the same time, it can use the theoretical advantages of the fusion distribution itself to suppress the interference of the observation noise on the proposed distribution without adding additional computational load. The improved importance density function combines the advantages of L2 and L1 norm estimation. Then the resampling combines particle fission smoothing weights to alleviate particle degradation and sample impoverishment. The vehicle experiment of multi-source cooperative navigation shows that compared with EKF, RCFPF improves RMS, Accuracy, and Precision by 26.12%, 30.67%, and 18.46%; compared with CPF, it increases by 45.68%, 27.22%, and 56.85% in RMS, Accuracy, and Precision, respectively, compared with STPF, the RMS, Accuracy, and Precision are increased by 39.56.%, 29.76%, and 47.85% respectively, which achieves the theoretical optimization effect and further improves the accuracy and robustness of the particle filter. The experimental results confirm the superiority of the proposed algorithm, provide a new system state estimation strategy for the multi-source cooperative position, and also provide a way of thinking for the further improvement of the particle filter performance.

## Data availability 

The datasets generated during and/or analysed during the current study are available from the corresponding author on reasonable request.
